# Harnessing brassinosteroid signaling in rice: from molecular pathways to environmentally adaptive breeding

**DOI:** 10.3389/fpls.2026.1781641

**Published:** 2026-02-25

**Authors:** Shuai Li, HanJing Sha, JinHui Zhang, ZhongHua Wei, LiChao Liu, LongNan Men, ZhongHua Sun, TianPeng Zong, ZhiJun Cheng, ShuPeng Xie

**Affiliations:** 1 Heilongjiang Academy of Agricultural Sciences, Suihua, Heilongjiang, China; 2 State Key Laboratory of Crop Gene Resources and Breeding, Institute of Crop Sciences, Chinese Academy of Agricultural Sciences, Beijing, China; 3 Nanfan Research Institute, Chinese Academy of Agricultural Sciences, Sanya, China

**Keywords:** brassinosteroid, environmentally adaptive breeding, hormone cross-talk, rice, signaling pathway

## Abstract

Brassinosteroids (BRs), a class of steroidal phytohormones, play crucial roles in regulating plant growth, development, and environmental adaptation in rice. With the elucidation of BR signaling pathways in *Arabidopsis*, the rice BR regulatory network has been progressively uncovered, revealing both conserved and unique features. In this review, we first summarize recent advances in understanding BR signaling mechanisms in rice. We then focus on how BR signaling integrates environmental cues, including nutrient availability, high-temperature stress, drought stress, and pathogen responses, to fine-tune growth and yield. Moreover, we highlight the complex cross-talk between BR signaling and other phytohormones that enables dynamic responses to environmental fluctuations. Finally, we discuss the potential applications of BR-related genes in rice production, together with the challenges of their translation into practical agricultural systems, providing perspectives and opportunities for environmentally adaptive breeding. Taken together, recent discoveries deepen our understanding of BR signaling in rice and provide a conceptual framework for exploring its roles in environmentally adaptive growth regulation and crop improvement.

## Introduction

Brassinosteroids (BRs), the sixth class of plant hormones identified after gibberellins, ethylene, auxins, abscisic acid, and cytokinins, play pivotal roles in plant growth and development. They regulate fundamental cellular processes such as cell division, elongation, and differentiation, and control organ-level traits including root development, hypocotyl elongation, leaf morphogenesis, and chlorophyll biosynthesis. BRs also influence male gamete viability, leaf senescence, and enhance plant tolerance to both biotic and abiotic stresses, underscoring their importance in plant adaptation and productivity (Yang et al., 2011; Nolan et al., 2020; Tong and Chu, 2018; Yang et al., 2024; Vukasinovic et al., 2025). In rice, BRs regulate a wide range of yield-related traits, such as plant height (Niu et al., 2022), grain size (Qiao et al., 2017), leaf inclination (Zou et al., 2023), tiller number (Fang et al., 2020), and panicle grain number (Guo et al., 2025), suggesting their potential application in improving rice yield. In addition to directly regulating yield-related traits, BRs also modulate the environmental adaptability of rice. Nitrogen (N) and phosphorus (P) are essential nutrients for rice growth. Previous studies have reported that nitrogen can influence the biosynthesis and function of plant hormones (Chen et al., 2022). With recent advances, increasing evidence has revealed a close interplay between nitrogen and BRs in the coordinated regulation of rice growth and development (Liu et al., 2021a; Zhang et al., 2023). Previous studies have shown that phosphorus deficiency reduces leaf inclination in rice, a typical trait regulated by BRs in determining plant architecture (Tong and Chu, 2018; Ruan et al., 2018). Subsequent research further revealed that key proteins involved in the phosphate starvation response interact with central components of the BR signaling pathway, thereby integrating BR signaling with phosphate starvation signaling. When exposed to environmental stresses, rice also adjusts its growth and developmental processes by modulating BR signaling, thereby enhancing its ability to cope with adverse conditions (Yan et al., 2024). These studies indicate that BR signaling is closely linked with nutrient and environmental cues. In a broader context, plant growth and development are orchestrated by a complex hormonal network. BRs extensively crosstalk with other phytohormones, such as gibberellins (GAs), auxins (IAA), cytokinins (CTK), jasmonic acid (JA), Strigolactone (SL), and abscisic acid (ABA), to fine-tune diverse developmental processes and stress responses (Yin et al., 2025). Therefore, this review highlights recent advances in BR signaling in rice, the crosstalk of BRs with environmental cues and other phytohormones, and their potential applications in rice breeding.

## An overview of BR signaling in rice

**Figure 1:**
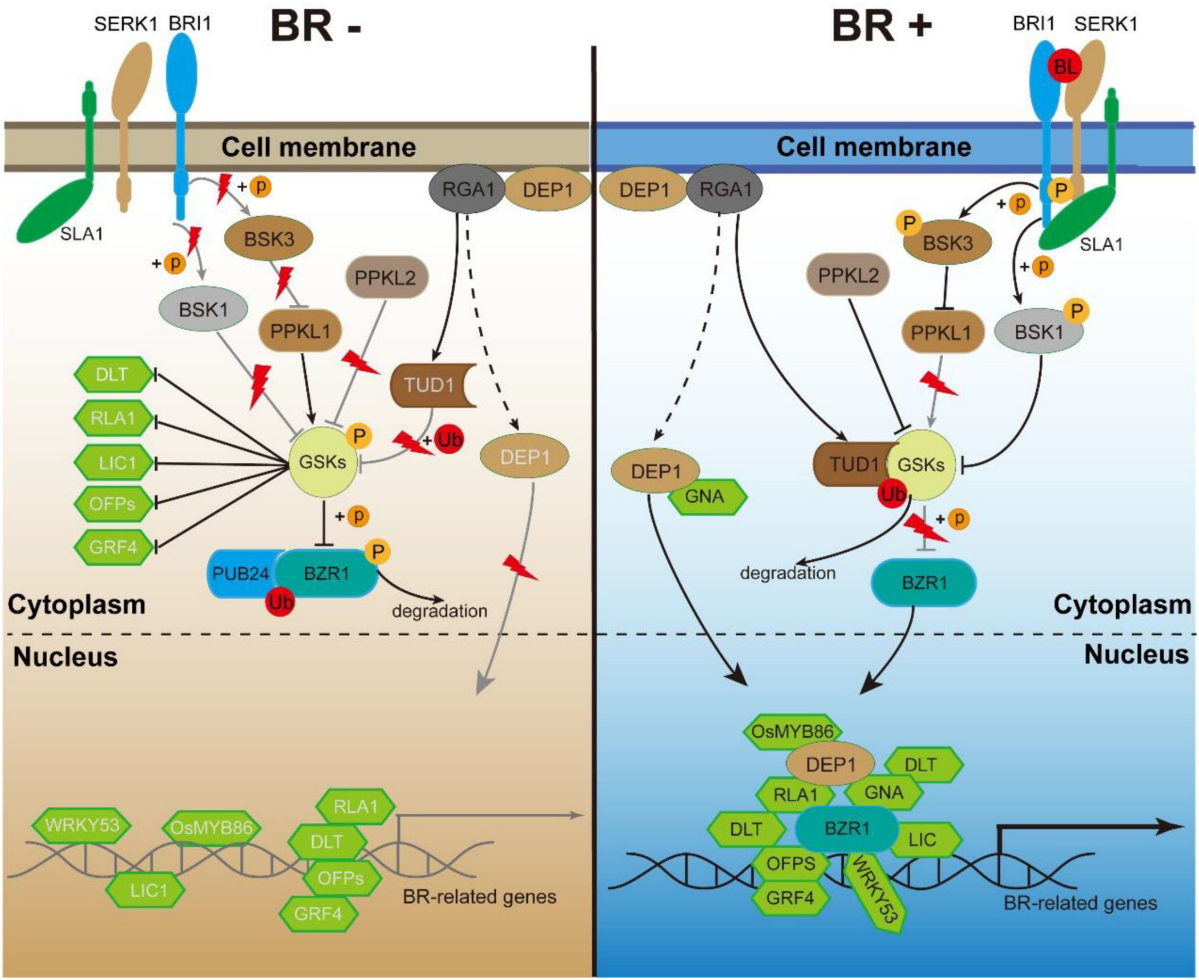
Overview of the BR signaling pathway in rice. Under BR-deficient conditions (left), the plasma membrane-localized receptor BRI1 remains inactive, preventing activation of BSKs and PPKL2 and leaving GSKs uninhibited. At the same time, TUD1-mediated ubiquitination of GSK2 is suppressed, enhancing its stability. The stabilized, active GSKs phosphorylate multiple BR-responsive transcription factors, including BZR1, reducing their stability and activity and thereby repressing BR signaling. Conversely, under BR-sufficient conditions (right), activation of the BRI1-SERK1 receptor complex leads to phosphorylation of BSKs, inhibition of PPKL1, and activation of PPKL2, promoting GSK inactivation. Simultaneously, TUD1 ubiquitinates GSK2, decreasing its stability. As a result, BR-responsive transcription factors are stabilized, accumulate in the nucleus, and enhance BR signal transduction. Arrows indicate activation or promotion, blunt-ended lines indicate inhibition, dashed arrows denote indirect or multi-step regulation, and “+p” or “+Ub” represent phosphorylation or ubiquitination, respectively.

In 1979, Grove et al. isolated 4 mg of the most active BR, brassinolide (BL), from 227 kg of rapeseed (*Brassica napus*) pollen (Mitchell et al., 1970; Grove et al., 1979), representing a major breakthrough in BR research. Over the following decades, extensive studies in *Arabidopsis* identified many genes involved in BR biosynthesis and signaling, thereby establishing a relatively complete regulatory framework. Building on this foundation, the subsequent cloning of BR signaling genes in rice revealed a signaling network that is broadly conserved with that of *Arabidopsis*, while also incorporating rice-specific components (**Figure 1**). In rice, BRs are perceived by the receptor kinase BRASSINOSTEROID-INSENSITIVE1 (BRI1) and its co-receptor SERK-family receptor-like protein kinase1 (SERK1)/BRI1-ASSOCIATED RECEPTOR KINASE (BAK1), whose kinase activities are activated upon BR binding (Yamamuro et al., 2000; Park et al., 2011). SMALL LEAF ANGLE 1 (SLA1) further strengthens their interaction, thereby promoting BR signal transduction (Song et al., 2022). Rice contains multiple BSKs, PPKLs, and GSKs, whose functions are not entirely redundant. Notably, GSKs are homologous to *Arabidopsis* BRASSINOSTEROID INSENSITIVE2 (BIN2), a glycogen synthase kinase 3/SHAGGY-like kinase, with both GSK2 and GSK3 functioning as repressors of BR signaling in rice (Li and Nam, 2002; Tong et al., 2012; Gao et al., 2019). *qGL3* encodes phosphatase with Kelch-like repeat domain1 (PPKL1), which dephosphorylates GSK3 to enhance its stability, thereby negatively regulating BR signaling and grain length (Gao et al., 2019). PPKL2, another Kelch-like repeat domain phosphatase, acts in the opposite manner by dephosphorylating GSK2, reducing its kinase activity and consequently promoting BR signaling and grain length (Yan et al., 2024). BRASSINOSTEROID-SIGNALING KINASE 1 (BSK1) can be phosphorylated by BRI1 and positively transmits BR signaling by inhibiting GSK2-mediated phosphorylation of BRASSINAZOLE RESISTANT1 (BZR1) (Tian et al., 2023). BRASSINOSTEROID-SIGNALING KINASE2 (BSK2) also interacts with BRI1 and is thought to function through homodimerization or heterodimerization with BRASSINOSTEROID-SIGNALING KINASE 3 (BSK3) and BRASSINOSTEROID-SIGNALING KINASE 4 (BSK4) (Yuan et al., 2022). BSK3 is phosphorylated by BRI1 and enhances BR signaling by suppressing the OsPPKL1-mediated dephosphorylation of OsGSK3 (Zhang et al., 2016; Tian et al., 2022). Together, these BSKs act as key positive regulators that relay signals from the BRI1 receptor complex to downstream components in the rice BR signaling pathway.

Downstream of GSK2, the transcription factor BZR1 serves as a central positive regulator of BR signaling. Under BR-deficient conditions, BZR1 is phosphorylated by GSKs in the cytoplasm. Phosphorylated BZR1 is excluded from the nucleus and targeted for degradation via PUB24-mediated ubiquitination, thereby impairing the regulation of target genes and repressing BR signaling. Under BR-sufficient conditions, GSK activity is inhibited, leading to BZR1 dephosphorylation. Dephosphorylated BZR1 accumulates in the nucleus, where it resumes its transcriptional activity and activates BR signaling (Bai et al., 2007; Tong et al., 2012). In addition to BZR1, GSK2 interacts with several other transcription factors, including DWARF AND LOW-TILLERING (DLT) (Tong et al., 2012), SMALL ORGAN SIZE1 (SMOS1)/REDUCED LEAF ANGLE1 (RLA1) (Qiao et al., 2017), TILLER ANGLE INCREASED CONTROLLER1 (LIC1) (Zhang et al., 2012), OVATE FAMILY PROTEIN (OFPs) (Yang et al., 2016), and GROWTH-REGULATING FACTOR4 (GRF4) (Che et al., 2015), to suppress their transcriptional activity and thereby inhibit BR signaling. Other transcription factors, such as WRKY53 (Tian et al., 2017; Liu et al., 2021b; Tian et al., 2021), OsMYB86 (Li et al., 2025), and DWARF AND LOW-TILLERING2 (DLT2)/GRAIN NUMBER ASSOCIATED (GNA) (Zou et al., 2023), also act as positive regulators of BR signaling.

Beyond the canonical BR signaling pathway, accumulating evidence indicates that heterotrimeric G-protein components also contribute to BR signaling. The Gα subunit DWARF1 (D1) has been shown to participate in BR responses, as loss-of-function *d1* mutants exhibit reduced sensitivity to BR (Wang et al., 2006). The Gγ subunit DENSE AND ERECT PANICLE1 (DEP1) interacts with GNA/DLT2, which facilitate its translocation from the cytoplasm to the nucleus. In the nucleus, DEP1 interacts with BR-related transcription factors and modulates their transcriptional activity, thereby enhancing BR signaling (Zhang et al., 2024b; Li et al., 2025; Wang et al., 2024). TAIHU DWARF1 (TUD1) encodes an E3 ubiquitin ligase that transduces BR signaling through direct interaction with the Gα subunit D1. Moreover, TUD1 physically interacts with GSK2 and promotes its ubiquitination-dependent degradation, thereby relieving the repression of BR signaling (Hu et al., 2013; Liu et al., 2023). Collectively, these results highlight the pivotal role of heterotrimeric G-protein components in mediating BR signaling.

MAPK signaling pathways have been shown to interact with various signaling networks to regulate multiple processes related to plant growth and development in rice. A typical MAPK cascade consists of at least three kinases: a MAPK kinase kinase (MKKK), a MAPK kinase (MKK), and a MAPK (Zhang and Zhang, 2022b; Xu and Zhang, 2015). Previous studies have reported that the OsMKKK10–OsMKK4–OsMAPK6 cascade regulates grain size in rice (Xu et al., 2018), and MAPK signaling cascades are also known to play crucial roles in controlling leaf angle (Wang et al., 2025a), which is a classic BR-regulated phenotype. In recent years, the relationship between MAPK signaling and BR pathways has begun to be elucidated. For example, MKK4/SMALL GRAIN 1 (SMG1) influences BR responses and the expression of BR-related genes. As a downstream component of the cascade (Duan et al., 2014), MAPK6 loss-of-function mutants exhibit reduced sensitivity to BR (Liu et al., 2015). Furthermore, MAPK6 can phosphorylate WRKY72, enhancing its transcriptional activation of *BRI1* and thereby promoting BR signaling (Wang et al., 2025a). These findings indicate that the MAPK signaling pathway engages in a direct crosstalk with the BR signaling pathway.

A growing body of evidence indicates that multiple (b)HLH family proteins function as critical regulators of BR signaling. Previous studies have demonstrated that INCREASED LEAF INCLINATION1 (ILI1) and ILI1 BINDING bHLH (IBH1) antagonistically regulate brassinosteroid (BR) signaling and rice leaf inclination. More recent evidence indicates that ILI1 interacts with BRASSINOSTEROID UPREGULATED1 (BU1)/ILI4, promoting the nuclear translocation of BU1. Together, ILI1 and BU1 suppress the transcriptional activity of IBH1, thereby enhancing BR signaling (Li et al., 2025). OsbHLH92 acts upstream by activating *BU1* transcription and positively contributes to the D1-mediated BR signaling pathway (Teng et al., 2023). Furthermore, ILIs form complexes with OsbHLH157/OsbHLH158 to fine-tune BR responses, thereby maintaining signaling homeostasis (Liu et al., 2024). ATBS1-INTERACTING FACTOR 1 (OsAIF1/OsbHLH176) and OsAIF2/OsbHLH178 act synergistically and redundantly to negatively regulate rice leaf inclination and grain size. In addition, both OsAIF1 and OsAIF2 physically interact with OsbHLH92 and antagonize its activity, thereby modulating rice development and downstream gene transcription (Lu et al., 2024). Collectively, these findings highlight (b)HLH proteins as key nodes that integrate BR signaling with transcriptional regulation. Based on the above, we summarized BR-related transcription factors, including their families and main functions, to provide a systematic overview of the BR signaling network and to guide future studies on their roles in rice growth and environmental adaptation (**Table 1**).

**Table 1:** Functionally characterized transcription factors involved in BR signaling in rice.

Gene	Locus	TF family	Function	Reference
BZR1	LOC_Os07g39220	BZR1/BES1 family	Core BR transcription factor interacting with GSK2 and regulating BR-responsive gene expression	(Qiao et al., 2017; Bai et al., 2007)
DLT/SMOS2	LOC_Os06g03710	GRAS protein	GSK2-interacting protein; key component of transcriptional complex in BR signaling	(Tong et al., 2009, 2012; Hirano et al., 2017)
DLT2/GNA	LOC_Os03g51330	GRAS protein	BZR1-interacting protein; key component of transcriptional complex in BR signaling	(Zou et al., 2023; Zhang et al., 2024b)
GRF4/GL2	LOC_Os02g47280	GROWTH-REGULATING FACTOR family	Downstream of BZR1; BR-responsive	(Che et al., 2015; Duan et al., 2015)
ILI1	LOC_Os04g54900	atypical bHLH protein	Direct downstream of BZR1; BR-responsive; HLH/bHLH complex component in BR signaling	(Zhang et al., 2009a; Liu et al., 2024)
ILI4/BU1	LOC_Os06g12210	atypical bHLH protein	BR-responsive; HLH/bHLH complex component in BR signaling	(Liu et al., 2024; Tanaka et al., 2009; Li et al., 2025)
ILI15/BUL1	LOC_Os02g51320	atypical bHLH protein	HLH/bHLH complex component in BR signaling	(Jang et al., 2017; Liu et al., 2024)
IBH1	LOC_Os04g56500	bHLH protein	Direct downstream of BZR1; BR-responsive; HLH/bHLH complex component in BR signaling	(Zhang et al., 2009a; Li et al., 2025)
LIC1	LOC_Os06g49080	CCCH-Type Zinc Finger Protein	Suppresses BZR1 expression; antagonizes BZR1 function	(Zhang et al., 2012; Duan et al., 2023)
MIR396d	LOC_Os04g57830	MicroRNA	Direct downstream of BZR1; BR-responsive;	(Tang et al., 2018)
OsARF11	LOC_Os04g56850	auxin response factor	Transcriptional regulation of BRI1; BR–auxin crosstalk	(Sakamoto et al., 2013; Li et al., 2025)
OsbHLH176	LOC_Os03g19780	atypical bHLH protein	HLH/bHLH complex component in BR signaling	(Lu et al., 2024)
OsbHLH92	LOC_Os09g32510	bHLH protein	HLH/bHLH complex component in BR signaling	(Lu et al., 2024; Teng et al., 2023)
OFP1	LOC_Os01g12690	Ovate Family Protein	Direct downstream of BZR1; BR-responsive;	(Xiao et al., 2017)
OFP3	LOC_Os01g53160	Ovate Family Protein	GSK2-interacting protein; BZR1-interacting protein; key component of transcriptional complex in BR signaling	(Xiao et al., 2020)
OFP8	LOC_Os01g64430	Ovate Family Protein	GSK2-interacting protein in BR signaling	(Yang et al., 2017)
OFP19	LOC_Os05g25910	Ovate Family Protein	GSK2-interacting protein; Key component of transcriptional complex in BR signaling	(Yang et al., 2018; Huang et al., 2022)
OsMYB86	LOC_Os01g50720	MYB family transcription factor	Component of transcriptional complex in BR signaling	(Li et al., 2025)
RLA1/SMOS1	LOC_Os05g32270	AP2-Type Transcription Factor	Key component of transcriptional complex in BR signaling	(Hirano et al., 2017)
WRKY53	LOC_Os05g27730	WRKY transcription factor	Key component of transcriptional complex in BR signaling	(Tian et al., 2017, 2021)
WRKY72	LOC_Os11g29870	WRKY transcription factor	Transcriptional regulation of BRI1	(Wang et al., 2025a)

## Crosstalk between BR signaling and environmental factors

**Figure 2:**
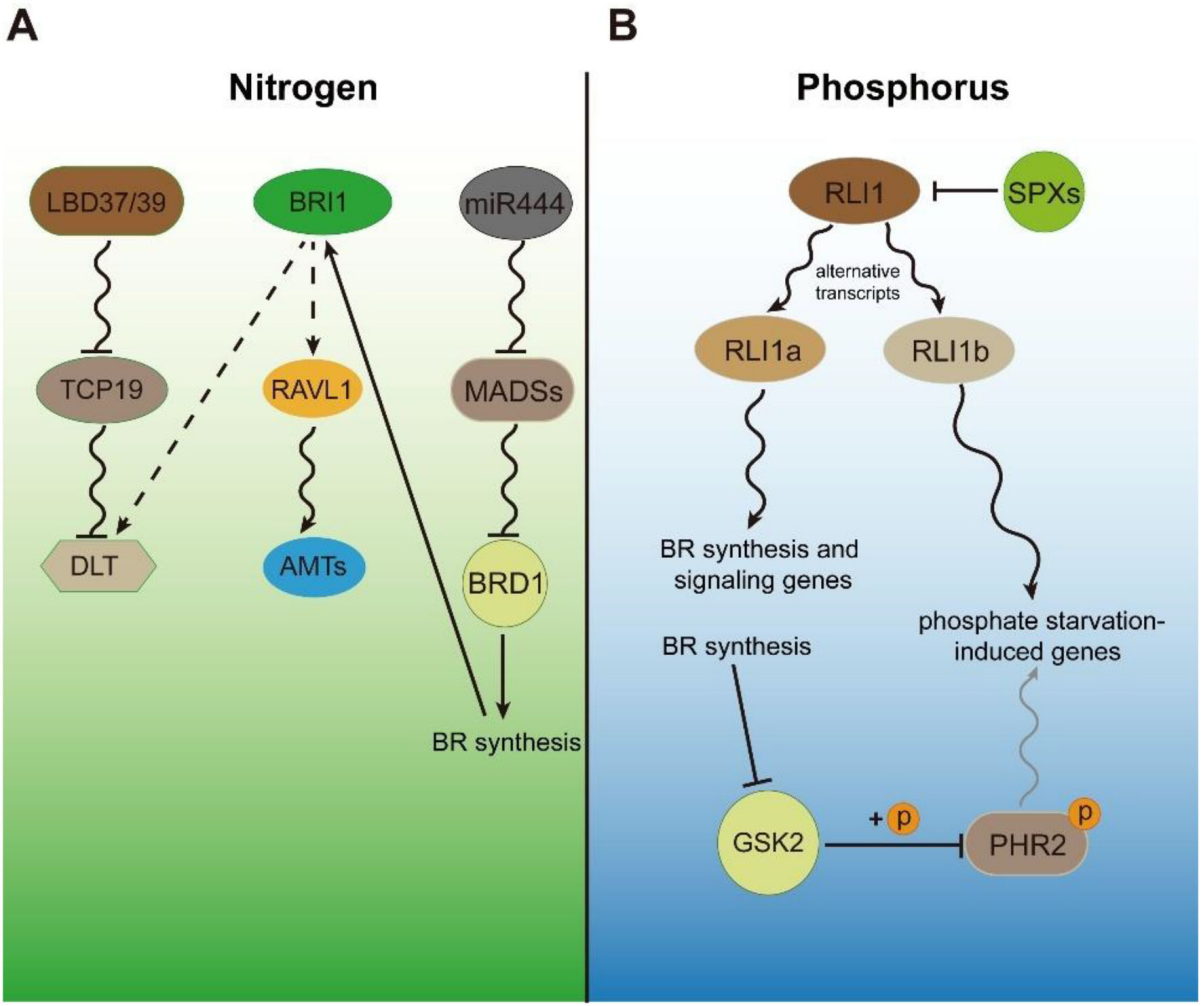
Crosstalk between BR signaling and nitrogen and phosphorus in rice. **(A)** Pure ammonium promotes the accumulation of miR444, which enhances BR biosynthesis by relieving the transcriptional repression of BRD1 mediated by several MADS-box proteins. Elevated endogenous BR levels activate RAVL1 through the BRI1-dependent signaling pathway, leading to transcriptional activation of AMTs and enhanced ammonium uptake in rice roots. DLT functions as an integrative regulatory node in both BR signaling and nitrogen-response pathways to control tillering. The nitrogen-response negative regulator LBD suppresses *TCP19* expression, while TCP19, as a transcription factor, represses *DLT* expression, forming a regulatory module that fine-tunes rice tiller development. **(B)** Pi starvation induces SPX1/2, which interact with RLI1 to inhibit its transcriptional activity. *RLI1* undergoes alternative splicing to generate two isoforms, *RLI1a* and *RLI1b*. RLI1a, but not RLI1b, directly activates BR biosynthesis and signaling genes, while both isoforms participate in Pi starvation responses. Meanwhile, BR signaling promotes phosphate uptake by attenuating GSK2-mediated phosphorylation of PHR2. Arrows indicate activation or promotion, blunt-ended lines indicate inhibition, and dashed arrows denote indirect or multi-step regulation. The curve represents transcriptional regulation, while “+P” or “+Ub” represent phosphorylation or ubiquitination, respectively.

Nitrogen (N) is the most essential macronutrient required for rice growth and development, and its availability is a key determinant of grain yield. In agricultural soils, N is predominantly present as nitrate (NO_3_^-^), ammonium (NH_4_^+^), or organic nitrogen. Among these, rice roots preferentially absorb the inorganic forms, nitrate and ammonium. The balance and dynamics of these inorganic N species are strongly influenced by soil oxygen status and microbial processes (Nasholm et al., 2009; Zhang et al., 2019). When soil oxygen levels are high, nitrate content is elevated, and rice predominantly takes up nitrate through nitrate transporters (NRTs). Conversely, under flooded or acidic conditions, ammonium becomes the dominant form of nitrogen, and rice primarily absorbs it via ammonium transporters (AMTs) (Li et al., 2017). Nitrogen use efficiency is closely associated with rice tiller number, and brassinosteroids (BRs) are also key regulators of tillering, implying potential crosstalk between nitrogen and BR signaling (Liu et al., 2021a; Tong et al., 2012, 2009). Nitrogen enhances the transcription and protein abundance of NITROGEN-MEDIATED TILLER GROWTH RESPONSE5 (NGR5), which interacts with RICE LEAF INCLINATION2 (LC2) to recruit the POLYCOMB REPRESSIVE COMPLEX 2 (PRC2). This complex mediates H3K27me3 deposition, repressing the target genes *DWARF14* (*D14*) and IDEAL PLANT ARCHITECTUTRE1 (IPA1), thereby promoting nitrogen-induced tiller formation in rice (Wu et al., 2020). Notably, NGR5 is the same protein as the previously identified BR signaling transcription factor SMOS1/RLA1 (Qiao et al., 2017), offering direct evidence that BR signaling participates in the nitrogen response in rice. A genome-wide association study identified *TCP19* as a nitrogen-efficiency gene that functions as a transcriptional repressor of tillering. A 29-bp deletion in the upstream regulatory region of TCP19 was shown to underlie varietal differences in nitrogen-responsive tillering. In nitrogen-efficient varieties carrying this deletion, the nitrogen-responsive repressor LATERAL ORGAN BOUNDARIES DOMAIN (LBD) protein binds efficiently to the locus, thereby suppressing *TCP19* expression. Reduced TCP19 activity leads to de-repression of the BR-signaling gene *DLT*, ultimately enhancing tiller development (Liu et al., 2021a). These findings elucidate the molecular mechanism of nitrogen-mediated tillering and provide evidence for an interaction between nitrogen and BR signaling pathway. Ammonium induces the expression of miR444, which promotes BR biosynthesis by relieving the repression of *BRD1* exerted by its MADS-box protein targets. Through activation of the miR444-MADS-BRD1 signaling cascade, ammonium enhances BR biosynthesis and consequently strengthens BR signaling, leading to the inhibition of root elongation (Jiao et al., 2020). Beyond regulating BR biosynthesis and signaling, BR also feeds back to modulate ammonium uptake. Acting downstream of BRI1, RELATED TO ABI3/VP1-LIKE 1 (RAVL1) directly binds to the *AMT1;2* promoter and activates its transcription, thereby enhancing root ammonium absorption (Xuan et al., 2017; Je et al., 2010) (**Figure 2A**). Together, these findings reveal a bidirectional regulatory relationship, in which nitrogen availability influences BR synthesis and signaling, while BR signaling in turn modulates nitrogen uptake, underscoring their coordinated roles in rice growth and development.

In addition to nitrogen, phosphorus (Pi) is another essential macronutrient for rice growth and yield. Despite its overall abundance in soils, Pi is often one of the most limiting nutrients for rice, largely because orthophosphate (inorganic phosphate, Pi)—the preferred form taken up by plants—has low solubility and a high tendency to be adsorbed onto soil particles (Schachtman et al., 1998). Therefore, the ability to adapt to low-phosphate conditions, as well as the maintenance of phosphate homeostasis and signaling, is particularly crucial for rice growth, development, and yield formation. One of the most striking phenotypes of phosphate (Pi) deficiency in rice is the appearance of erect, spindly leaves with reduced tiller numbers, which closely resembles the phenotypes caused by impaired BR biosynthesis or signaling. These morphological similarities suggest a potential link between Pi starvation signaling and BR signaling. In maintaining phosphate homeostasis and signaling, PHOSPHATE STARVATION RESPONSE (PHR) proteins and SPX domain-containing proteins (SPXs) play pivotal roles (Puga et al., 2017). In rice, phosphate starvation signaling induces the expression of SPX1/2, which interact with REGULATOR OF LEAF INCLINATION1 (RLI1) to inhibit its transcriptional activation of *BU1* and *BC1*. As BU1 is a key transcription factor in the BR signaling pathway that positively regulates leaf angle, this repression ultimately results in a reduced leaf angle under phosphate-deficient conditions (Ruan et al., 2018). Subsequent studies revealed that RLI1 undergoes alternative splicing, producing two isoforms: RLI1a, which contains a MYB DNA-binding domain, and RLI1b, which contains both MYB and coiled-coil (CC) domains. The absence of a CC domain in RLI1a allows it to activate a broader set of target genes compared to RLI1b. Notably, RLI1a, but not RLI1b, directly regulates both BR biosynthesis and signaling by activating BR-related genes. Both isoforms, however, participate in modulating phosphate (Pi) starvation signaling (Guo et al., 2022). Recent studies have also shown that under phosphate-starvation conditions, RLI1a exhibits reduced inhibition of the E3 ubiquitin ligase PUB77. The resulting accumulation of PUB77 promotes the ubiquitination of BZR3, thereby attenuating BR signaling and ultimately altering rice plant architecture (Wang et al., 2025b). Conversely, BR signaling can feedback to regulate phosphate uptake: the BR kinase GSK2 phosphorylates and suppresses the transcriptional activity of the central Pi regulator PHR2, while Pi starvation can reduce GSK2 stability, forming a BR-mediated transcriptional pathway that promotes phosphate absorption (Zhang et al., 2024a) (**Figure 2B**). These results indicate a reciprocal regulation between phosphate availability and BR signaling: Pi starvation affects BR biosynthesis and signaling, whereas BR activity feeds back to modulate phosphate homeostasis and uptake, emphasizing their integrated role in rice growth and adaptation.

**Figure 3:**
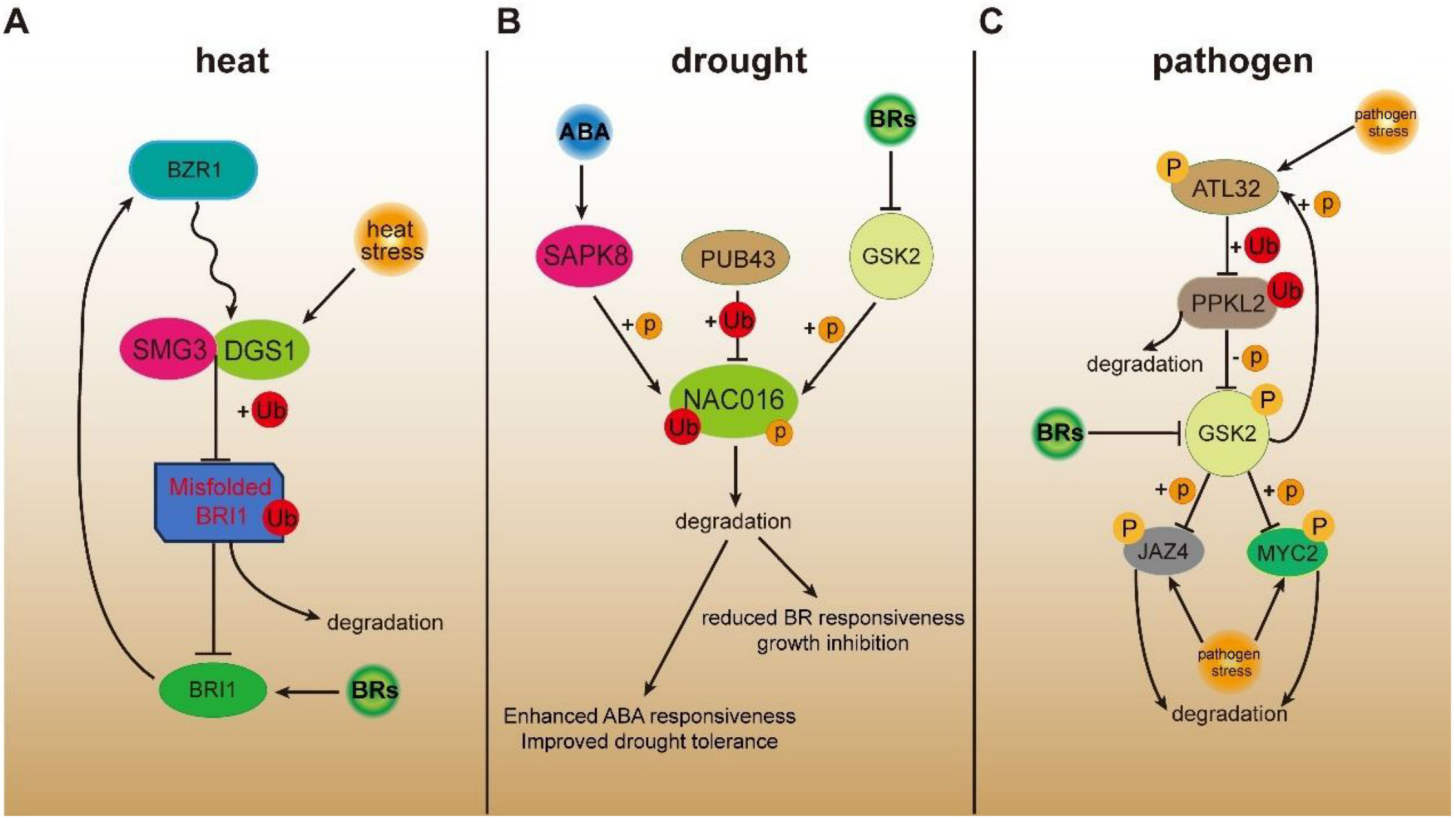
Crosstalk between BR signaling and environmental stress in rice. **(A)** BZR1 directly activates *DGS1/TT3.1*, enhancing rice thermotolerance under high-temperature conditions. The SMG3–DGS1/TT3.1 complex mediates the ubiquitination and degradation of misfolded or incompletely folded BRI1, thereby preserving normal BRI1 signaling integrity. **(B)** When BR levels increase, BR inhibits GSK2-mediated phosphorylation of NAC016, stabilizing the protein, promoting BR responses, and suppressing ABA responses. Conversely, when ABA levels increase, ABA enhances SAPK8-mediated phosphorylation of NAC016, leading to PUB43-dependent ubiquitination and 26S proteasome degradation, thereby suppressing growth and enhancing drought tolerance. **(C)** ATL32 targets the BR signaling component PPKL2 for ubiquitination-mediated degradation, attenuating both BR signaling and rice blast resistance. PPKL2 interacts with and dephosphorylates GSK2 to promote immunity, while GSK2 phosphorylates and stabilizes ATL32, reinforcing its negative role in blast resistance. GSK2 has been reported to phosphorylate JAZ4, triggering COI1-dependent degradation and enhancing antiviral defense; however, other studies indicate that GSK2 promotes OsMYC2 degradation, thereby suppressing JA-mediated defense and facilitating viral infection. Arrows indicate activation or promotion, blunt-ended lines indicate inhibition, and dashed arrows denote indirect or multi-step regulation. The curve represents transcriptional regulation, while “+P” or “+Ub” represent phosphorylation or ubiquitination, respectively.

In addition to regulating plant growth and development, BRs also play important roles in plant responses to diverse biotic and abiotic stresses. With the intensification of global warming, extreme high temperatures have emerged as a major threat to global food security. Through large-scale screening and thermotolerance phenotyping of 22,762 rice accessions, a novel QTL, TT3, was identified and cloned, which confers high-temperature tolerance in rice. The TT3 locus comprises two antagonistic genes, *THERMO-TOLERANCE3.1* (*TT3.1*) and *THERMO-TOLERANCE3.2* (*TT3.2*), that positively and negatively regulate thermotolerance, respectively. The plasma membrane–localized E3 ubiquitin ligase TT3.1 enhances rice heat tolerance by alleviating chloroplast damage caused by the accumulation of TT3.2 under stress (Zhang et al., 2022a). Notably, *TT3.1* maps to the same locus as *DECREASED GRAIN SIZE1* (*DGS1*), a previously characterized BR signaling gene. OsBZR1 directly binds to the *DGS1*/*TT3.1* promoter and activates its expression, thereby positively regulating grain size (Zhu et al., 2021). *SMALL GRAIN 3* (*SMG3*) encodes an E2 ubiquitin-conjugating enzyme and interacts with DGS1/TT3.1 to form the SMG3-DGS1 ubiquitination complex (Li et al., 2023). The SMG3-DGS1/TT3.1 complex mediates the ubiquitination of misfolded or incompletely folded BRI1, promoting its degradation. Loss of function of either SMG3 or DGS1/TT3.1 results in the accumulation of misfolded or incompletely folded BRI1, which interferes with the normal function of BRI1 and consequently leads to reduced grain size in rice (Li et al., 2023) (**Figure 3A**). These results indicate that DGS1/TT3.1 plays a crucial role in both rice thermotolerance and BR signaling, suggesting that DGS1/TT3.1 may serve as a key node through which BRs mediate rice adaptation to high-temperature stress.

When environmental temperatures rise, plants may experience not only heat stress but also drought stress. Previous studies have reported that BR treatment can enhance plant drought tolerance (Kagale et al., 2007; Krishna, 2003). However, other studies have shown that BR-deficient mutants, which exhibit semi-dwarf stature and erect architecture, also display improved drought resistance (Beste et al., 2011; Ferrero-Serrano and Assmann, 2016; Zolkiewicz and Gruszka, 2024), indicating that BR plays a highly complex role in balancing plant growth and development with drought tolerance. In *Arabidopsis*, *RESPONSIVE TO DESICCATION26* (*RD26*) encodes a NAC-family transcription factor. RD26 can interact with the BES1 protein, antagonizing BES1’s transcriptional activity on BR-regulated genes. Conversely, BR signaling represses the expression of RD26 and its homologs, thereby suppressing drought responses (Ye et al., 2017). In rice, loss of function of the G-protein α subunit DWARF 1 (D1) reduces the plant’s sensitivity to BR (Wang et al., 2006). After 14 days of drought treatment, *d1* mutant leaves remained dark green and erect, whereas wild-type leaves became yellowed and wilted. The *d1* mutant exhibited higher stomatal conductance and lower stomatal limitation to photosynthesis, while transpiration rates were similar to the wild type. This difference may be attributed to variations in leaf surface temperature between the two genotypes (Ferrero-Serrano and Assmann, 2016). A recent study indicates that BR-mediated regulation of the balance between growth and drought tolerance in rice is closely linked to ABA, with NAC016 acting as a key node in BR-ABA crosstalk. When BR levels increase, BR suppresses GSK2-mediated phosphorylation of NAC016, enhancing NAC016 protein stability, which promotes BR responses, inhibits ABA responses, and favors growth at the expense of drought tolerance. Conversely, when ABA levels increase, ABA promotes SAPK8-mediated phosphorylation of NAC016, facilitating PUB43-mediated ubiquitination and 26S proteasome degradation of NAC016, thereby suppressing growth while enhancing drought tolerance (Wu et al., 2022) (**Figure 3B**). Taken together, studies in rice indicate that BR promotes growth while reducing drought tolerance, whereas in Arabidopsis, BR exhibits more diverse effects on drought responses. Whether the role of BR in regulating drought tolerance is species-specific or dynamically linked to plant growth and development remains to be further investigated. Corresponding to drought stress, cold stress is another major environmental challenge. In rice, studies on the regulatory relationship between BR and cold stress are still limited, but some evidence indicates that BR can enhance cold tolerance, whereas the BR-insensitive mutant *d61-1* (loss of BRI1 function) shows reduced cold tolerance (Cheng et al., 2024). The downstream molecular mechanisms underlying BR-cold stress crosstalk remain to be further elucidated.

In rice, activation of immune responses during growth often compromises yield, whereas suppression of immunity promotes rapid growth but increases pathogen susceptibility. Maintaining a balance between growth and immunity involves the coordinated regulation of numerous genes. Notably, BR signaling components such as SERK2 (Chen et al., 2014), BSK1 (Wang et al., 2017), and BZR1 (Yuan et al., 2023) have been shown to play key roles in modulating rice immune responses. ATL32 encodes an E3 ubiquitin ligase that targets the BR signaling component PPKL2 for ubiquitination and 26S proteasome-mediated degradation, thereby attenuating rice blast resistance and BR signaling. Conversely, PPKL2 interacts with and dephosphorylates GSK2 to promote immunity, while GSK2 stabilizes and activates ATL32 through phosphorylation, reinforcing its negative role in blast resistance (Yan et al., 2024). Beyond BR signaling, GSK2 also participates in JA-mediated defense. It has been reported that GSK2 phosphorylates JASMONATE ZIM-DOMAIN4 (JAZ4), triggering CORONATINE INSENSITIVE1 (COI1)-dependent degradation and enhancing antiviral defense (He et al., 2020), whereas another study showed that GSK2 promotes OsMYC2 degradation, thereby suppressing JA-mediated defense and facilitating viral infection (Hu et al., 2020) (**Figure 3C**). These opposing findings suggest that BR may not function as an exclusive antiviral hormone but rather act as a context-dependent regulator whose role in immunity could be influenced by crosstalk with other hormonal pathways. Further studies are required to unravel the precise mechanisms underlying these diverse outcomes.

## Crosstalk between BR signaling and other phytohormones in rice

Accumulating evidence suggests that BR rarely acts in isolation; rather, it functions through coordination or antagonism with other plant hormones, such as abscisic acid (ABA), jasmonic acid (JA), gibberellins (GA),auxin, cytokinins (CTK), and strigolactones (SL).These hormones not only interact with BR but also influence each other, collectively forming a complex and dynamic regulatory network (Depuydt and Hardtke, 2011). To facilitate a clearer understanding of BR-mediated regulation, we present the interactions between BR and each individual hormone separately, instead of depicting all hormone interactions simultaneously.

**Figure 4:**
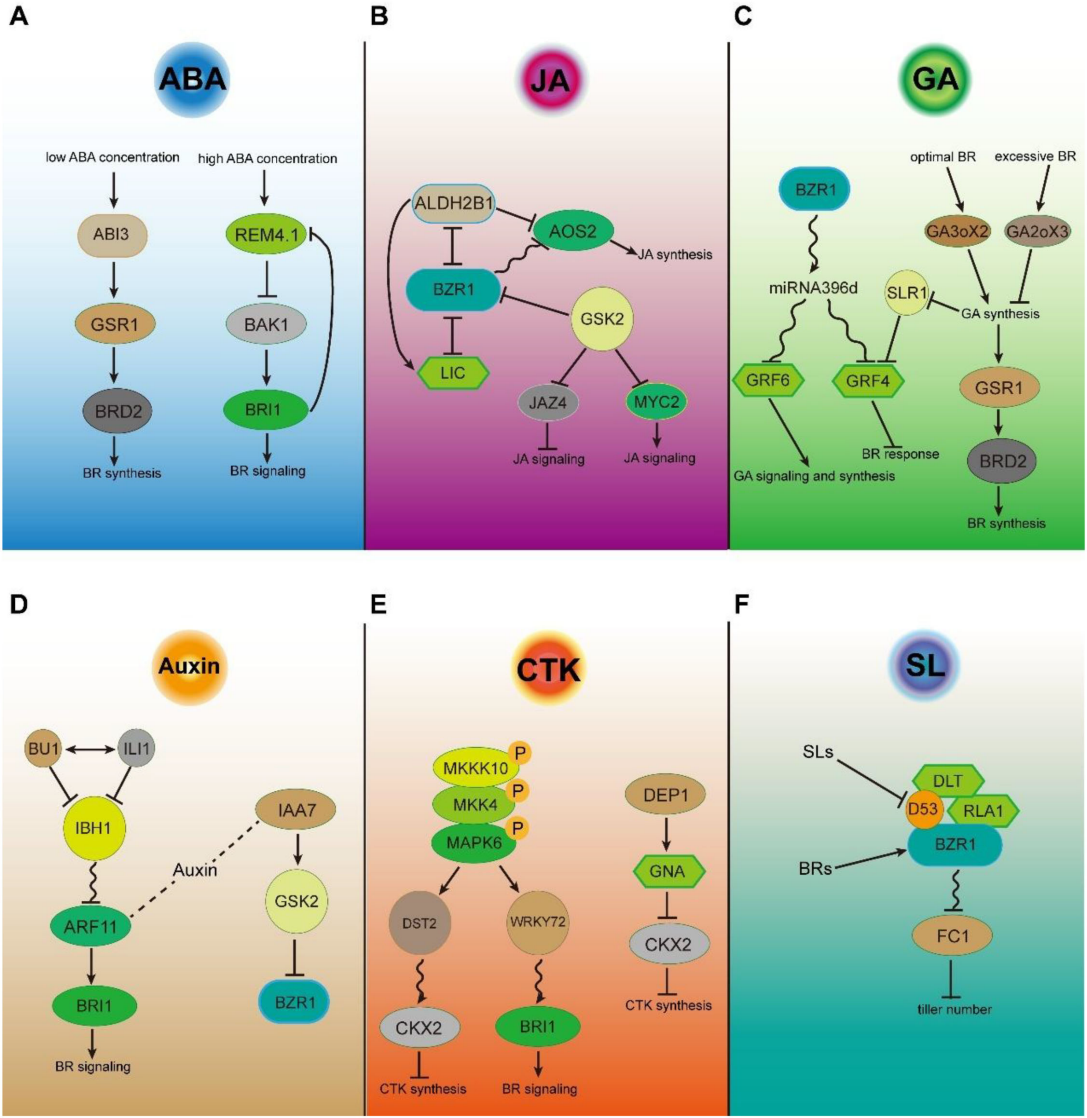
Crosstalk between BR signaling and other phytohormones in rice. Schematic illustration of the crosstalk between BR signaling and other plant hormone pathways, including **(A)** abscisic acid (ABA), **(B)** jasmonic acid (JA), **(C)** gibberellins (GA), **(D)** auxin, **(E)** cytokinins (CTK), and **(F)** strigolactone (SL). Arrows indicate activation or promotion, blunt-ended lines indicate inhibition, and dashed arrows denote indirect or multi-step regulation. The curve represents transcriptional regulation, while “+P” or “+Ub” represent phosphorylation or ubiquitination, respectively.

As classical stress hormones, abscisic acid (ABA) and jasmonic acid (JA) play central roles in plant stress adaptation, and their extensive crosstalk with brassinosteroid (BR) signaling constitutes a core regulatory module underlying BR-mediated environmental adaptability in plants. Abscisic acid (ABA), a central regulator of stress responses, and brassinosteroids (BRs), key promoters of plant growth, are generally regarded as antagonistic hormones in *Arabidopsis*. Their interplay modulates multiple developmental and physiological processes, including seed germination, stomatal closure, root growth, and early seedling development (Zhang et al., 2009b). In addition to NAC016 acting as a regulatory node integrating ABA and BR signals to balance rice growth and drought tolerance (Wu et al., 2022), recent studies indicate that ABA-BR interactions during rice growth and development can be either synergistic or antagonistic, depending on ABA concentration. At low levels, ABA transiently activates BR signaling, in part through GSR1, a BR biosynthesis regulator induced by ABI3. This short-term activation also enhances ABA-mediated salt stress tolerance. By contrast, high ABA levels rapidly induce *REM4.1*, which suppresses BR signaling (Li et al., 2021) (**Figure 4A**). Together, these findings underscore the dual roles of ABA in modulating BR pathways and deepen our understanding of their crosstalk. Jasmonic acid (JA) is a well-established regulator of plant defense against a broad range of pathogens, including bacteria, fungi, and viruses (Major et al., 2017). In rice, BR-JA crosstalk appears to play an important role in defense responses. ALDH2B1 and BZR1 function antagonistically and may form a complex that mutually inhibits each other’s DNA-binding activity. *ALDH2B1* and *AOS2* are direct targets of BZR1, which represses their expression, while ALDH2B1 and LIC also suppress *AOS2* transcription. Suppression of ALDH2B1 and LIC activates JA biosynthesis and signaling, thereby enhancing rice defense (Ke et al., 2020). This provides evidence that BR can modulate rice defense through JA. In addition, GSK2 may contribute to antiviral defense by regulating the activity of MYC3 or JAZ4, although this mechanism remains to be clarified (**Figure 4B**).

GAs, as key phytohormones in rice, play crucial roles in regulating both growth and development as well as environmental adaptation. GA and BR both contribute significantly to the control of plant height, grain size, and leaf inclination in rice. Under normal physiological conditions, moderate levels of BR can elevate GA levels in rice by inducing the expression of the GA biosynthesis gene *GA3ox2*, thereby promoting cell elongation and increasing plant height. Under excessive BR conditions, GA levels in rice are reduced through *GA2ox3*-mediated inactivation, thereby suppressing plant height (Tong et al., 2014). The expression of *GSR1* (GAST family gene in rice1) is induced by GA but repressed by BR. By interacting with the BR biosynthetic enzyme DIM/DWF1, GSR1 activates BR biosynthesis. Thus, GSR1 plays important roles in both BR and GA pathways and serves as a mediator of the crosstalk between these two signaling pathways (Wang et al., 2009). GA can also modulate BR responses in rice. GRF4, a key component of GA signaling, interacts with SLR1, and GA promotes SLR1 degradation, thereby enhancing GRF4 transcriptional activity. OsMIR396d, a direct target of the transcription factor OsBZR1, participates in BR signaling, likely by repressing the growth-regulating factorGRF4 to influence BR responses. OsMIR396d can affect GA signaling and GA biosynthesis by suppressing GRF6, thereby contributing to the regulation of plant height (**Figure 4C**). Furthermore, DLT physically interacts with ORYZA SATIVA HOMEOBOX (OSH15) to regulate rice internode elongation through the BR signaling pathway (Niu et al., 2022). OSH15 can directly bind to the promoters of *GA2ox3*, *GA2ox5*, *GA2ox8*, and *GA2ox9*, strongly repressing their expression to modulate GA levels in rice (Zhang et al., 2024c). These findings suggest that GRF4 and OSH15 may function as critical node genes mediating the crosstalk between GA and BR signaling.

The phytohormones auxins and brassinosteroids are both essential regulators of physiological and developmental processes, and it has been suggested that they act inter-dependently and synergistically. In rice, auxin co-application improves the brassinosteroid response in the rice lamina inclination bioassay. In addition, the auxin response factor ARF11 can directly bind to the promoter of *BRI1* and activate its expression. Within the BR signaling pathway, the (b)HLH transcription factors BU1, ILI1, and IBH1 collectively regulate the transcription of *ARF11*. Recent studies further showed that AUXIN or INDOLE-3-ACETIC ACID 7 (IAA7) interacts with GSK2, thereby enhancing GSK2-mediated phosphorylation and degradation of BZR1, which negatively regulates BR signaling (**Figure 4D**). Although this finding uncovers a role of IAA7 in BR signaling, its function in auxin signaling remains unclear. Thus, IAA proteins may mediate the crosstalk between BR and auxin, a possibility that warrants further investigation.

Plant hormones, particularly cytokinins (CTKs), are well known for their roles in rice panicle morphogenesis (Kieber and Schaller, 2018). Recent studies have also shown that the BR biosynthesis gene *BRD3* and the BR signaling gene *BRI3* regulate grain number per panicle (Zhang et al., 2024d; Guo et al., 2025). In addition, the SP3–DEP1–APO2 module likely regulates both BR and CTK signaling to control panicle architecture (Liu et al., 2025). These results suggest that BR and CTK might function together in regulating grain number per panicle in rice. OSH15 modulates internode elongation by interacting with DLT to regulate *BRI1* expression (Niu et al., 2022). In addition, OSH15 can directly bind to the promoter of the cytokinin oxidase gene *CKX4*, repressing its transcription, thereby reducing cytokinin degradation and influencing the development of tillers, spikelets, and glumes (Wang et al., 2022). MAPK6, as a downstream component of the MAPK cascade, can function as a regulatory node integrating cytokinin and BR signaling. It reduces cytokinin biosynthesis by enhancing DST-mediated transcriptional activation of *CKX2* (Guo et al., 2020, 2018), while simultaneously promoting BR signaling by enhancing WRKY72-mediated transcriptional activation of *BRI1* (Wang et al., 2025a), suggesting that MAPK6 coordinates the crosstalk between cytokinin and BR pathways. GNA positively regulates grain number by binding to the promoter of *CKX2* and repressing its expression (Zhang et al., 2024b) (**Figure 4E**). GNA also functions as a BR signaling component (DLT2) and regulates BR signaling through interactions with DLT and BZR1 (Zou et al., 2023). These findings indicate that OSH15 and GNA may function as key nodes in the crosstalk between cytokinins and BR signaling.

Strigolactones (SLs), a group of terpenoid lactones phytohormone, have been recently identified to strongly inhibit bud outgrowth in different plant species, which is indicated by more tillers in the rice mutants involved in SL biosynthesis and signaling. Recent studies indicate that BR signaling promotes rice tillering by stimulating bud outgrowth. Both SL and BR pathways appear to regulate tillering through the stability of DWARF53 (D53) or the OsBZR1–RLA1–DLT transcriptional complex within the BR signaling pathway. Evidence suggests that D53 interacts with OsBZR1 to suppress the expression of FINE CULM1 (FC1), a local inhibitor of tillering (**Figure 4F**). This repression depends on the ability of OsBZR1 to directly bind the *FC1* promoter and recruit D53 to rice buds, highlighting a coordinated mechanism by which BR signaling modulates tiller development (Fang et al., 2020).

Overall, these studies indicate that during crosstalk between BR signaling and other hormones in rice, certain key proteins act as nodal points, functioning both in the BR pathway and in the biosynthesis or signaling of other hormones. Such nodal genes likely serve as important mediators of hormone-hormone interactions. Some proteins even participate in multiple hormonal pathways simultaneously; for instance, SHORT INTERNODES1 (SHI1) is involved in BR biosynthesis and signaling, ABA responses, and auxin biosynthesis (Duan et al., 2023). The existence of these proteins provides a molecular basis for the formation of a more intricate hormone regulatory network in rice.

## Implications of BR signaling for environmentally adaptive rice breeding

**Figure 5:**
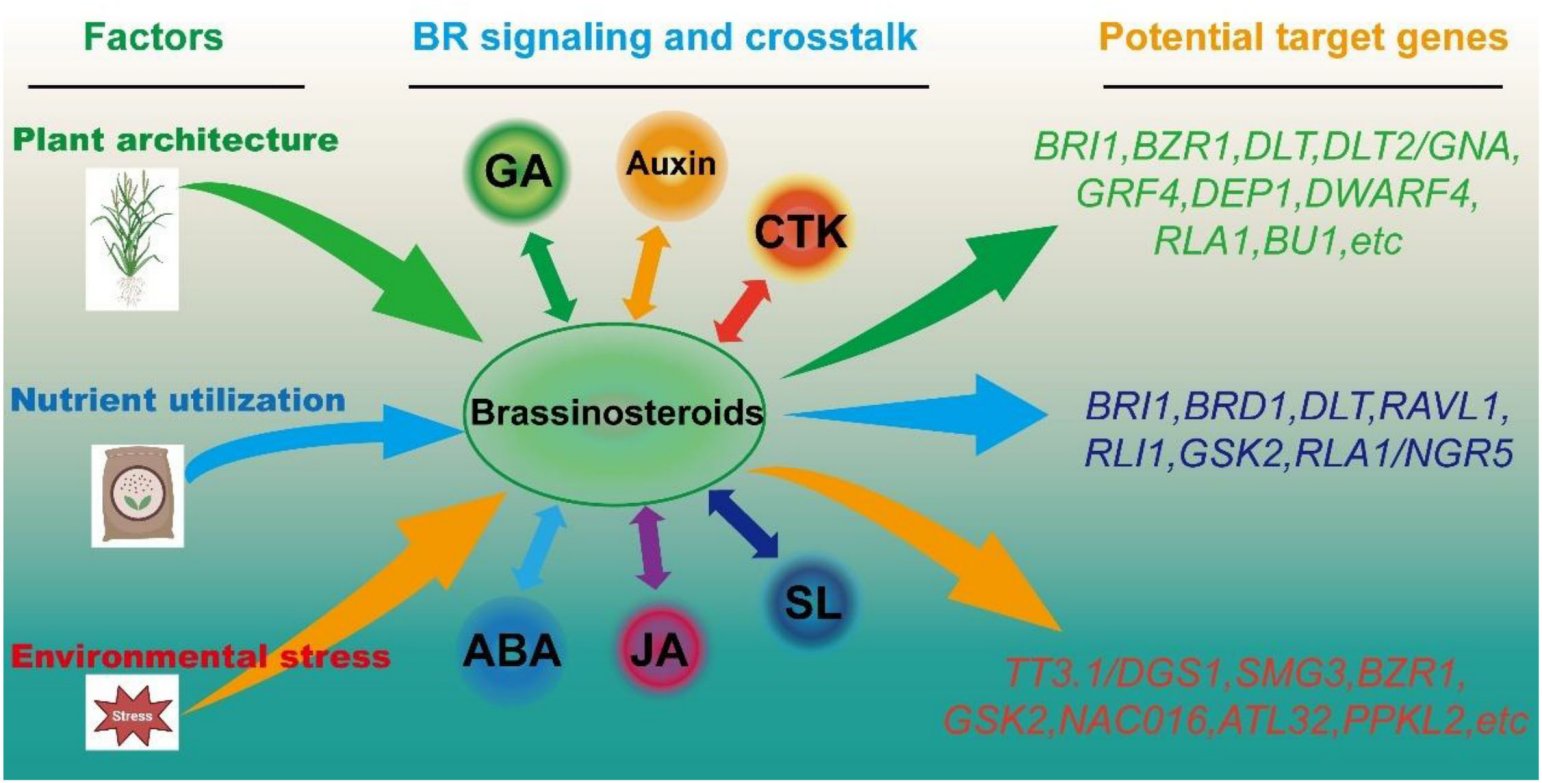
BR-related genes for environmentally adaptive rice improvement. This schematic summarizes BR-related genes involved in key adaptive traits, including plant architecture optimization, nutrient use efficiency, and environmental stress resistance. The diagram integrates major BR signaling components (e.g., *BRI1*, *DLT*, *DLT2/GNA*, *BZR1*, *GRF4*, *DEP1*, *DWARF4*, *RLA1/NGR5*, *BU1*, *TT3.1/DGS1*, *ALT32*, etc) and highlights their potential contributions to environmentally adaptive rice improvement.

Environmental change imposes increasing constraints on rice productivity through temperature extremes, drought, salinity, and nutrient imbalance. In addition, large amounts of chemical fertilizers and pesticides are applied annually to protect rice from pests and diseases, increasing agricultural input costs and causing environmental pollution, which conflicts with the principles of green, eco-friendly, and sustainable agriculture. Brassinosteroids (BRs) play crucial roles in integrating internal developmental processes with external environmental cues in plants, offering a potential strategy to address both rice production and environmental challenges. First, BRs have significant potential to enhance the efficiency of light and nutrient utilization, serving as a key support for environmentally adaptive breeding. Notably, BRs exert a unique influence on leaf angle, a trait closely associated with canopy architecture, planting density, and light interception. Rice plants with erect leaves allow for higher planting density and improved photosynthetic efficiency, ultimately contributing to enhanced grain yield. Most BR-deficient mutants in rice exhibit erect leaves and semi-dwarf phenotypes; for example, the BR biosynthetic mutant *dwarf4* shows improved yield performance under high-density cultivation (Sakamoto et al., 2006). However, this yield advantage is context-dependent, being observed specifically under high-density planting conditions, where *dwarf4* outperforms the wild type at the same planting density. Therefore, this represents a conditional yield advantage rather than a general yield increase under normal field conditions. Moreover, the application of other BR-related genes to improve rice plant architecture and planting density remains limited. In contrast, tissue-specific expression or targeted editing of BR-related genes represents a feasible and promising strategy to avoid pleiotropic effects. For example, secondary branch meristem–specific activation of *BRD3* increases spikelet number without causing detrimental whole-plant phenotypes (Zhang et al., 2024d). Similarly, in maize, the gene *LEAF ANGLE ARCHITECTURE OF SMART CANOPY 1* (*LAC1*) generates a “smart canopy” architecture with an erect upper canopy and a looser lower canopy, without accompanying negative traits, thereby enhancing light-use efficiency and increasing yield potential under dense planting (Tian et al., 2024). These studies provide valuable insights and possibilities for improving rice plant architecture, increasing planting density, and ultimately boosting grain yield. Nitrogen and phosphorus are fundamental nutrients that underpin rice productivity, and improving their use efficiency is critical both for sustaining yields and for reducing the environmental burden of fertilizer overuse. Emerging evidence shows that brassinosteroid (BR) signaling is tightly intertwined with the plant’s responses to these two nutrients. On the nitrogen side, BR signaling integrates with nitrogen status at multiple levels: nitrogen availability can influence tillering through the BR regulator DLT, whereas BR perception via BRI1 feeds back to modulate ammonium uptake (Liu et al., 2021a; Je et al., 2010; Xuan et al., 2017). Ammonium itself reinforces this connection by promoting BR biosynthesis through BRD1 (Jiao et al., 2020), creating a regulatory loop that shapes overall plant growth. Phosphorus interacts with BR signaling in a similarly multilayered manner. Phosphate starvation responses mediated by SPXs, RLI1, and PUB77 can fine-tune the transcription of BR-related genes, thereby adjusting growth under phosphate limitation (Ruan et al., 2018; Guo et al., 2022; Wang et al., 2025b). At the same time, BR signaling can enhance phosphate acquisition by acting through GSK2 to dampen PHR2 activity and activate downstream phosphate starvation–inducible genes (Zhang et al., 2024a). Together, these findings suggest that manipulating BR signaling components involved in nitrogen and phosphorus pathways—such as DLT, BRI1, BRD1, and GSK2—offers a promising approach to improve nutrient use efficiency. Incorporating these alleles into elite germplasm could contribute to the development of environmentally adaptive rice varieties that achieve high yields with reduced fertilizer dependency and lower environmental impact. Beyond nutrient-related constraints, increasingly frequent extreme environmental events pose additional challenges to rice production. Notably, the BR signaling gene *DGS1* and the major heat-tolerance locus *TT3.1* are the same gene, raising the possibility that targeted editing of BR pathway genes could enhance thermotolerance in rice (Zhang et al., 2022a; Li et al., 2023). Although the role of BRs in antiviral immunity remains unclear, accumulating evidence suggests a close connection between BR signaling and rice responses to viral pathogens, a relationship that warrants further investigation (Hu et al., 2020; He et al., 2020). The growing body of research also highlights the remarkable complexity of BR signaling and its extensive crosstalk with other hormonal pathways. Interactions between BRs and ABA or JA are particularly intriguing (He et al., 2020; Gui et al., 2016; Li et al., 2021), given the central roles of these hormones in mediating stress adaptation. Whether BR-mediated environmental resilience operates independently of ABA and JA, or relies on coordinated signaling with these pathways, remains an open question. Although direct editing of BR signaling genes has the potential to enhance rice tolerance to various environmental stresses, such modifications often result in undesirable pleiotropic effects on growth and development. Recent work, however, offers a promising way to resolve this trade-off: tissue-specific activation of *BRD3* in secondary branch meristems can increase panicle branching while avoiding penalties on grain size, ultimately boosting grain yield (Zhang et al., 2024d). These findings suggest that tissue-specific expression or editing of BR-associated genes may provide an effective strategy to improve environmental adaptability while minimizing negative impacts on plant architecture and overall performance. Based on the above, we provide an integrative schematic conceptual framework highlighting BR-related candidate genes potentially involved in plant architecture, nutrient use, and stress adaptation, thereby addressing the proposed “environmentally adaptive rice” perspective (**Figure 5**).

## Challenges and limitations of BR-related modifications

Despite the promising potential of BR signaling in rice improvement, several limitations and uncertainties remain. First, the effects of manipulating the same BR-related gene may differ across genetic backgrounds, because BR functions through extensive crosstalk with multiple hormones and signaling pathways. Genes located at the intersection of BR and other pathways may exhibit multifunctional roles, leading to non-uniform phenotypes in different cultivars. For example, GSK2 has been reported to participate in JA-mediated defense, but with contrasting outcomes: it phosphorylates JAZ4, triggering COI1-dependent degradation and enhancing antiviral defense (He et al., 2020), whereas it promotes OsMYC2 degradation, suppressing JA-mediated defense and facilitating viral infection (Hu et al., 2020). Whether these discrepancies are due to differences in genetic background remains to be investigated. A related issue is whether BR-related mutants or engineered lines maintain stable performance under complex field conditions. Unlike controlled laboratory experiments, real agricultural environments involve simultaneous stresses such as drought, heat, and pathogen pressure, and it is currently unclear whether BR-related modifications can consistently confer desired phenotypes under such multifactorial conditions. Therefore, although BR-related genes hold significant potential for rice improvement, their practical application requires careful evaluation, multi-environment validation, and context-specific strategies to ensure stability and avoid unintended trade-offs.

## Conclusions and perspectives

Over the past decades, intensive studies have greatly deepened our understanding of brassinosteroid (BR) signaling, establishing a relatively well-defined regulatory network in both *Arabidopsis* and rice. Numerous BR-related genes have been cloned and characterized, underscoring their pivotal roles in regulating rice growth and development. Beyond these developmental functions, accumulating evidence highlights the importance of BRs in rice adaptation to diverse environmental conditions. Notably, BRs do not act in isolation but rather coordinate with other plant hormones to fine-tune growth, development, and stress resilience. In particular, *TT3.1/DGS1* and *NGR5/RLA1* provide representative examples illustrating practical strategies for deploying BR-related genes or allelic variants in elite germplasm. Notably, both genes function not only within BR signaling, but also participate in multiple regulatory pathways, underscoring the integrative nature of BR-mediated regulation. Under high-temperature conditions, overexpression of *TT3.1/DGS1* has been reported to increase rice yield by more than 2.5-fold (Zhang et al., 2022a; Li et al., 2023). Similarly, enhanced expression of *NGR5/RLA1* improves nitrogen use efficiency while maintaining the desirable semi-dwarf architecture and high-yield traits, thereby enabling higher yields under reduced nitrogen input conditions (Wu et al., 2020; Qiao et al., 2017). Together, these examples highlight the necessity of understanding BR crosstalk with other signaling networks and clearly demonstrate the practical breeding potential of harnessing BR signaling to enhance stress tolerance and nutrient efficiency in rice improvement programs.

Despite these advances, several key challenges remain in the field of rice BR signaling: (i) Identifying key regulatory nodes. A central task is to pinpoint the core genes that integrate BR signaling with environmental cues and other hormonal pathways. These regulatory nodes will be crucial for decoding how BR crosstalk coordinates rice growth, development, and stress adaptation. (ii) Engineering tissue-specific BR regulation. Although naturally occurring alleles with tissue-specific BR gene expression can mitigate detrimental whole-plant effects, relying solely on natural variation is insufficient. Instead, precision genome editing to achieve stable, tissue-specific regulation of BR-related genes will be essential for maximizing stress resilience while avoiding pleiotropic penalties. (iii) Resolving the spatial and developmental dynamics of BR action. BR levels fluctuate dynamically across developmental stages and differ substantially between adjacent tissues. Because hormone crosstalk is highly dose-dependent—and even a single hormone can trigger opposite responses at different concentrations—single-cell transcriptomics and proteomics will be invaluable for mapping the spatiotemporal activity of BR-related genes and proteins. Such high-resolution datasets will provide a theoretical foundation for rational, tissue-specific BR gene editing. (iv) Expanding the BR signaling network. Although the canonical BR pathway is well established in both *Arabidopsis* and rice, recent discoveries have revealed several noncanonical BR signaling routes, underscoring the pathway’s complexity. Continued exploration of these atypical components will refine our understanding of BR biology and open new avenues for applying BR signaling to rice improvement. Collectively, these developments indicate that BR research is entering a new phase—one that moves beyond basic mechanistic insights toward translational strategies for sustainable rice production and global food security.
